# Risk factors for hazardous drinking in university students from South Africa and Belgium: a cross-cultural comparison study

**DOI:** 10.4314/ahs.v21i1.17

**Published:** 2021-03

**Authors:** Yasemin Inaç, Ynke Larivière, Muhammad Hoque, Guido Van Hal

**Affiliations:** 1 University of Antwerp Faculty of Medicine and Health Sciences, Department of Epidemiology and Social Medicine; 2 University of Antwerp, Department of Epidemiology and Social Medicine; 3 Senior Research Associate, Management College of Southern Africa, Durban, South Africa

**Keywords:** Hazardous drinking, university students, South Africa, Belgium

## Abstract

**Background:**

Previous studies have associated certain risk factors with hazardous drinking in students. However, big cultural and geographical differences exist regarding alcohol use.

**Objectives:**

To determine whether or not there was a difference in hazardous drinking between Belgian and South African university students and to establish the risk factors that contribute to hazardous drinking in university students (calculated using the AUDIT-C) from a developing country (South Africa) and a developed country (Belgium).

**Methods:**

An online survey assessing hazardous drinking among university students in South Africa (University of KwaZulu-Natal, UKZN) and Belgium (University of Antwerp, UoA) was conducted, using the shortened version of the Alcohol Use Disorder Identification Test (AUDIT-C). Risk factors in males and females for hazardous drinking were explored using multivariate logistic regression analysis.

**Results:**

In total, 499 students were included in the study (250 UoA and 249 UKZN students). A significant higher amount of male (94.8%) as well as female (92.4%) UoA students drank alcohol in the last year compared to the male (66.2%) and female (67.8%) UKZN students (p<0.001). Additionally, a significant higher amount of UoA students were hazardous drinkers, compared to the UKZN students (p<0.001). Multivaiate analysis showed that male UoA students were almost 6 times more likely to be hazardous drinkers than male UKZN students (OR=5.611, p=0.005). Female UoA students were more than twice as likely to be hazardous drinkers than female UKZN students (OR=2.371, p=0.016).

**Conclusion:**

This study found a significant difference in hazardous drinking between Belgian and South African university students.

## Introduction

Nowadays, alcoholic beverages are an important part of students' social lives^1^. While occasional (<1.3 g ethanol per day) and low-volume drinking (1.3–24.9 g ethanol per day) have not been associated with higher morbidity and mortality risks^2^, increasing alcohol amounts can lead to negative consequences^2^–^4^. Particularly, college and university students have been known to drink more hazardously than the general population^3^, ^4^, leading to several health risks (such as psychosocial problems, personal injuries, traffic accidents, sexually transmitted infections, etc.) and decreased academic performances^5^–^8^. Previous studies have associated certain risk factors with hazardous drinking in students. These factors include demographic factors such as gender and age^9^, ^10^, socio-economic factors such as income and parental educational level^9^–^11^ and, lifestyle and educational related factors such as subjective health, physical activity, nutrition awareness, study-related stress and subjective health^12^–^15^. Furthermore, several studies found that ethnic groups differ in their alcohol consumption, whereby Caucasian students often consume more alcohol than Asian or Black students^16^–^19^. However, in general, European and North American students consume more alcohol than their counterparts from Asia and Africa^20^.

Every country has its own alcohol policy, the general principles of these policies can be found in the Alcohol Report from the World Health Organization (WHO), updated in 2018^4^. First of all, there are legal age differences to purchase alcohol. For example, in Belgium the minimum age for alcohol consumption depends on the type of alcohol. Adolescents (≥ 16 years old) are legally allowed to purchase wine and beer but to purchase spirits the minimum age is 18. However, not all countries differentiate according to age. For example, in South-Africa the minimum age to buy all types of alcoholic beverages is 18 years old^4^. Second of all, the maximum amount of alcohol which the government deems safe to consume on a weekly basis differs in the two countries. In Flanders, the government recently changed this policy and deems 10 glasses a week for both men and woman the maximum advisable limit of alcohol consumption^21^. At the moment, there is no guideline in South-Africa which states the amount of alcohol deemed safe. However, the government advices citizens to consume alcohol sensibly^22^.

In today's fast paced environment globalisation is increasing and international student exchanges are becoming more common. Since the majority of studies on students' alcohol consumption are focused on the United States^8^, ^23^, ^24^, it is uncertain if those results could be extrapolated to other developed and developing countries. Understanding the factors associated with alcohol use and their similarities and differences among student populations in different countries could open new paths for interventions. Furthermore, to the best of our knowledge, this study is the first one to measure alcohol use in both South African and Belgian students. Therefore, the aims of this study were to determine (i) whether or not there was a difference in hazardous drinking between Belgian and South African university students and, (ii) to determine the risk factors that contribute to hazardous drinking in university students (calculated using the AUDIT-C) from a developing country (i.e. South-Africa) and a developed country (i.e. Belgium).

## Methods

### Setting and study design

This study was a cross-sectional, cross-cultural, comparative study conducted among university students in South Africa and Belgium. More specifically, students from the University of KwaZulu-Natal (UKZN) in Durban and students from the University of Antwerp (UoA) in Antwerp were asked to take part in an online survey assessing substance use among university students.

### Questionnaire

The questionnaires used for this study were based on a Flemish survey that has been used to assess substance use in Flemish tertiary education students three times previously. The more general Belgian study, ‘In hogere sferen?’ (‘Head in the Clouds’ - HITC), has been conducted every four years since 2005^25^–^28^. Each time the study is repeated, the questionnaire is reassessed and updated by a panel of experts from various Belgian universities. For this cross-cultural study, the Belgian students were assessed using the newest Dutch version of the questionnaire from 2017 and the South African students were asked to fill in an English version of the questionnaire used in 2013. This 2013 questionnaire was translated from Dutch to English by Linguapolis, the University Language Institute of the UoA. Afterwards this translation was updated, in collaboration with experts from the UKZN, to suit the South-African context. Therefore, some minor differences were present in both questionnaires as upgrading and translating the 2013 Dutch questionnaire was done simultaneously to ensure both questionnaires could be implemented in the same time period. However, the small differences in questionnaires were not an issue as the alcohol related sections used for this study were conceptually equal in both questionnaires.

To assess the risk for alcohol use disorder, the shortened version of the Alcohol Use Disorder Identification Test (AUDIT), namely the AUDIT-C was included in both questionnaires. These questionnaires can be used to assess alcohol use disorder or hazardous drinking, which is an umbrella term for alcohol dependency, -intoxication and -abuse. Several previous studies have shown that the AUDIT is a valid instrument to use among students and that the AUDIT-C is an even better indicator for hazardous drinking by students^29^–^31^. Both the Dutch and English AUDIT and AUDIT-C have been validated^32^–^34^. The AUDIT-C consists of the following three questions: “How often do you drink alcohol?”, “If you drink alcohol, how many glasses do you usually drink per day?”, “How often do you drink 6 or more glasses of alcohol on a single occasion?”.

For male students a cut-off point of ≥ 5 was used for hazardous drinking and for female students a score of ≥ 4. These cut-off points were based on a previous study which stated that among a tertiary student population, in which heavy alcohol use is normative behaviour, less strict cut-off points for the AUDIT-C should be used to detect hazardous drinking behaviour^35^. Normally, the cut-off points are ≥ 3 for women and ≥ 4 for men^36^.

### Population and data collection

Sample size was calculated using G*Power software to determine how many male and female students had to be included in the study to find a possible significant difference in hazardous drinking among male and female students from both universities^37^. For the study to be adequately powered, 155 men and 138 women had to be included, if sensitivity was 80% given the expected prevalence of 58.9% hazardous drinking among men at the UoA, 32.8% of hazardous drinking among male UKZN students, 45.3% prevalence of hazardous drinking among female students of the UoA and 18.9% hazardous drinking among female UKZN students^7^, ^38^.

Primary data from the UKZN and secondary data (initially collected for the HITC-study) from the UoA. In the UKZN, data was collected from February until March 2018 using an online questionnaire which was sent via a weblink to the students' e-mail addresses. Data from the UoA, collected via a weblink for the HITC-study was collected from March until April 017. In total, 249 students from the UKZN and 2974 students from the UoA responded. To make the two groups comparable, a randomly selected sample of 250 students was drawn from the UoA students to compare to the 249 students from the UKZN. The sample size was sufficiently large according to the sample size calculation.

### Statistical analysis

Data analysis was conducted using SPSS version 25 and p-values of <0.05 were considered statistically significant. The internal consistency of the AUDIT-C was determined using Cronbach's alpha and a comparative analysis between both universities was performed for men and women with a Pearson chi-square test or Mann-Whitney U test depending on the characteristics of the data. To account for missing data, multiple imputation analysis was performed^39^.

Furthermore, using multivariate logistic regression, the risk factors assessed for hazardous drinking among male and female university students were (i) demographic variables such as university, age, students status, living situation, student club member, student club board member, sports club member and youth movement member and, (ii) substance use variables (i.e. substances used in the year previously to filling out the questionnaire) such as tobacco and cannabis use, types of alcohol consumed (wine, beer, liqueurs and hard liquors) and medication use (tranquilizers/sleeping medications, stimulants or illegal drugs). Backward elimination was used to achieve the final model. All variables with a p-value <0.15 were kept in the model, this is based on the knowledge that the traditional 0.05 level can fail to identify important variables^40^. Male and female students were analysed separately as several studies have shown a clear difference in their alcohol use and abuse^19^, ^24^, ^26^,^27^, ^41^–^43^.

## Ethical consideration

Ethical clearance certificates were obtained from the institutional review board at the University of Antwerp and the University of KwaZulu-Natal. Participation in the study was voluntary. Anonymity and confidentiality was maintained at all times.

## Results

### Internal consistency AUDIT-C

With an overall Cronbach's alpha of 0.797 there was a good internal consistency of the AUDIT-C. For the South African students, the Cronbach's alpha was 0.779 and 0.806 for the Belgian students.

### Sample characteristics

The sample characteristics, including the demographic characteristics and substance use (alcohol and others) are provided in table 1. The total sample included 499 university students, 163 were male and 336 were female. From the 163 males, 85 were students at the UoA and 78 were students at the UKZN. When looking at the demographic variables, the male students from the UoA were approximately 2 years younger than those attending the UKZN, were more likely to live at home (67.1% vs 29.2), more likely to have a temporary job (29.4% vs 15.4%) and less likely to be on a board of a student club (5.1% vs 15.4%). Other demographic characteristics were similar among both universities. When focussing on the alcohol use among male students, 94.8% of the UoA males drank alcohol in the past year, compared to 66.2% of the UKZN males. Furthermore, excluding those that didn't drink alcohol (n=30), the mean AUDIT-C is significantly higher among males from the UoA (5.39) than males from the UKZN (4.27). Additionally, more hazardous drinking was observed in UoA males than UKZN males (61.9% vs 28.2%). When looking at the types of alcohol consumed in the past year, males from the UoA drank significantly more beer, wine and hard liquors. No significant difference was observed in the consumption of liqueurs. Finally, no differences were observed in the use of other substances such as tobacco, cannabis, tranquilizrs or sleeping medication, stimulants and illegal drugs between males from both universities.

**Table 1:** Frequency table sample characteristics

	Men					Women				
	UoA (n= 85)		UKZN (n= 78)			UoA (n= 165)		UKZN (n= 171)		
	Yes (%)	No (%)	Yes (%)	No (%)	p (X^2^)	Yes (%)	No (%)	Yes (%)	No (%)	p (X^2^)
**Demographic variables**										

Gender	-	-	-	-	0.301	-	-	-	-	0.107
Age	Median: 21yo^a^		Median: 23yo^a^		**0.002** b	Median: 21yo^a^		Median: 22yo^a^		**<0.001** b
Student status (/week)										
- Non-working	64.7	-	80.8	-	**0.022**	47.3	-	65.0	-	**0.001**
- Temporary job (<20h)	29.4	-	15.4	-	**0.033**	43.6	-	25.5	-	**<0.001**
- Job (≥20h)	5.9	-	3.8	-	0.722^c^	9.1	-	9.5	-	0.904
Parental home	67.1	32.9	29.2	70.8	**<0.001**	61.8	38.2	53.2	46.8	0.114
Student club member	39.3	60.7	31.3	68.7	0.306	39.2	60.8	23.3	76.7	**0.002**
Student club board	5.2	94.8	15.4	84.6	**0.048** c	6.4	93.6	9.7	90.3	0.289
Sports club	39.3	60.7	24.4	75.6	0.051	31.4	68.6	13.7	86.3	**<0.001**
Youth movement	20.9	79.1	24.1	75.9	0.639	13.8	86.2	20.1	79.9	0.132

**Alcohol use**										

Total AUDIT-C	Mean: 5.39		Mean: 4.27		**0.018** d	Mean: 3.76		Mean: 2.63		**<0.001**
Drinking status	94.8	5.2	66.2	33.8	**<0.001**	92.4	7.6	67.8	32.2	**<0.001**
Hazardous drinkerse	61.9	38.1	28.2	71.8	**<0.001**	47.9	52.1	16.7	83.3	**<0.001**
Beer	88.7	11.3	65.4	34.6	**0.001**	75.6	24.4	21.6	78.4	**<0.001**
Wine	72.2	27.8	50.0	50.0	**0.004**	86.5	13.5	64.7	35.3	**<0.001**
Liqueurs	24.9	75.1	17.2	82.8	0.246	34.7	65.3	28.3	71.7	0.228
Hard liquors	82.1	17.9	55.1	44.9	**<0.001**	76.2	23.6	41.2	58.8	**<0.001**

**Other substance use**										

Tobacco	38.6	61.4	33.3	66.7	0.493	22.2	77.8	15.6	84.4	0.126
Cannabis	32.9	67.1	46.2	53.8	0.102	21.6	78.4	32.7	67.3	**0.028**
Tranquilizersf	5.9	94.1	6.9	93.1	0.656	10.8	89.9	8.8	91.2	0.554
stimulants	6.4	93.6	13.3	86.7	0.138	6.5	93.5	5.1	94.9	0.622
Illegal drugs	20.9	79.1	16.6	83.3	0.490	7.5	92.5	5.4	94.6	0.457

a yo = years als

b Mann-Whitney U test

c Fisher Exact test

d Student's t-test

e as calculated by the AUDIT-C

f or sleeping medication

Among the 336 female students, 165 attended the UoA and 171 attended the UKZN. Female students attending the UoA were approximately one year younger than the female students attending the UKZN (21 years old vs 22 years old). They also were more likely to have a temporary job (47.3% vs 25.5%), be a student club member (61.8% vs 53.2%) and be in a sports club (31.4% vs 13.7%). When looking at the differences in alcohol use in the past year, 92.4% of the UoA females drank alcohol compared to 67.8% of the UKZN females. Not only did they drink more, they also drank more hazardously (47.9% vs 16.7%). This can also be noticed when looking at the mean AUDIT-C which was 3.76 for UoA females and 2.63 for UKZN females, after excluding non-drinkers (n=67). Females from the UoA drank more beer, wine and hard liquors than females from the UKZN and no difference was found in the drinking of liqueurs. Finally, when looking at other substance use in the past year, the use of cannabis was significantly higher among UKZN females than among UoA females (32.7% vs 21.6%). No differences were observed in other substance use.

### Multivariate analysis

Table 2 and Table 3 show the final multivariate models for male and female university students, respectively. Multivariate analysis showed that male UoA students were almost 6 times more likely to be hazardous drinkers than male UKZN students (OR=5.611, 95% CI 1.719–18.414; p=0.005). Female UoA students were more than twice as likely to be hazardous drinkers than female UKZN students (OR=2.371, 95% CI 0.846–6.644; p=0.016). Other significant risk factors for hazardous drinking in males were living independently, being part of a youth movement or a board of a student club, drinking beer or hard liquors and smoking tobacco. For females, risk factors were being an older student, being part of a sports club, drinking wine or hard liquor, smoking tobacco or cannabis and using illegal drugs.

**Table 2:** Multivariate model males, Odds ratio's associated with male hazardous alcohol consumption

Variable	Adjusted OR	95% CI	*P*-value
University (UoA)	5.611	1.719–18.414	**0.005**
Living situation (independently)	3.342	1.040–10.736	**0.043**
Student club board	0.198	0.033–1.177	0.075
Youth movement	4.226	1.095–16.314	**0.037**
Beer	15.401	1.400–169.391	**0.025**
Hard liquor	20.092	3.564–113.261	**0.001**
Tobacco use	3.166	1.197–8.378	**0.020**

OR = Odds Ratio; CI = Confidence interval

**Table 3:** Multivariate model females, Odds ratio's associated with female hazardous alcohol consumption

Variable	Adjusted OR	95% CI	*P*-value
University (UoA)	2.371	0.846–6.644	**0.016**
Age	0.884	0.787–0.992	**0.037**
Sports club	2.484	1.013–6.095	**0.047**
Wine	5.532	1.356–22.559	**0.017**
Hard liquor	5.205	2.040–13.280	**0.001**
Tobacco use	2.629	1.151–6.001	**0.022**
Cannabis use	2.731	1.179–6.325	**0.019**
Illegal drug use	4.856	1.168–20.192	**0.030**

OR = Odds Ratio; CI = Confidence interval

## Discussion

The aims of this study were to determine whether or not there was a difference in hazardous drinking between Belgian and South African university students and to determine the risk factors that contribute to hazardous drinking in university students (calculated using the AUDIT-C) from a developing country (i.e. South-Africa) and a developed country (i.e. Belgium). Research on this topic is especially relevant since an increasing number of students participate in foreign exchanges, whereby they are not given adequate training on alcohol and drugs before going abroad^45^. Since university students already have an increased risk of harmful drinking and an adequate support system might be lacking abroad, research on this topic might guide services for university students in foreign countries. Results from this study indicate that both male and female students from UoA consume more alcohol compared to students from UKZN. The overall AUDIT-C score was also higher among male and female UoA students compared to students from UKZN. In line with this, the rate of harmful drinking among UoA students was higher compared to UKZN students. These findings are in line with results from previous studies which indicate that in most of Europe, less than 20% of the adult population abstains from alcohol ADDIN EN.CITE (WHO)2004117[4611711727World Health Organization (WHO)2004Geneva^45^, in contrasts, results from studies in Africa report abstention rates as high as 80% for women and 50% for men^46^. Furthermore, this difference might partially be explained by the difference in legal drinking age between Belgium and South Africa (i.e. 18 in South Africa versus 16 for beer and wines and 18 for spirits in Belgium) which could lead to a difference in drinking pattern between students.

As part of this study multivariate analyse was conducted to determine risk factors associated with hazardous drinking for males and female students. Among male students living independently was a risk factor for hazardous drinking, which corroborates an American study which found living away from one's parents either with peers or independently to be a risk factor for heavy drinking^47^. Being part of a youth movement was also found to increase the risk of harmful drinking for male students. This factor has not been linked to alcohol use among university students in previous studies, but it is plausible that youth movements share characteristics such as high-group cohesion and identity, easy availability of alcohol and a historical culture of heavy drinking with student clubs. Findings from previous studies suggest a strong relationship between student clubs and harmful drinking^48^–^50^. Membership of a youth movement as a risk factor for harmful alcohol consumption would, therefore, be plausible, but further research is needed to assess this relationship in-depth. Interestingly, membership of a student club was not found to be a risk factor for harmful alcohol use although previous research suggests a strong link between student club membership and harmful alcohol use^51^, ^52^. This might be explained by the relatively low rate of student club membership (25.8%) among UKZN students. Consumption of beer and hard liquor was also linked to an increased risk of harmful drinking. These findings are in line with results from a Belgian study which found an association between numerous negative consequences and drinking beer and spirits^7^. Smoking tobacco was also found to pose a risk for harmful drinking among male students. Previous studies have shown that there is a strong dependency on alcohol consumption and tobacco smoking to co-occur^53^, ^54^. Results also suggest that there is a higher vulnerability for alcohol use disorders among smokers of tobacco^55^. Smoking tobacco is also seen as a strong predictor for subsequent alcohol use, misuse and dependence^56^, ^57^.

On the other hand, factors associated with harmful drinking among female students differed from those found in males. An increase in age was found to associated with harmful drinking in female students. Which differs from previous studies that found a decrease in alcohol consumption when age increased among university students and that women under the legal drinking age consumed more alcohol compared to older women^11^,^58^. Membership of a sports club was also found to be a risk factor for harmful alcohol use among female students. Although there are many benefits attributed to sports, numerous studies found athletes to consume more alcohol, participate more frequent in binge drinking and heavy episodic drinking and experience negative consequences of alcohol use compared to their fellow students who did not participate in sports which are in line with our findings^59^–^61^. As with their male counterparts drinking hard liquor was associated with harmful alcohol use for female students. While instead of beer consumption for males, drinking wine was associated with harmful drinking for females. Similarly, to male students smoking tobacco also showed an increased risk for harmful drinking. But smoking cannabis and using other illegal drugs were also risk factors for female students. Which is interesting since numerous studies have recognized tobacco smoking and alcohol use to be early steps on the pathway to more extensive drug use^62^–^65^.

## Limitations

Due to the retrospective nature of the data several variables (e.g. race, religion etc.) that could influence the drinking behaviour of students were not collected in both questionnaires and could therefore not be assessed in the multivariate logistic regression. However, this might have been an impart gap as previous studies from South Africa have shown differences in alcohol consumption among students of different races, with white student drinking more hazardously than black students^66^. Secondly, another consequence to retrospective data collection was missing data. We approached the dataset with data “missing at random”. However, this issue was resolved by performing multiple imputation^67^. Using multiple imputation analysis may have led to a possible underestimation of substance use because the data miht have been “missing non at random” (e.g. a person might not have filled in a certain question because he/she was not willing to report his/her substance use)^68^. However, due to the long questionnaires it might also be possible that people gave up filling them in “completely at random”, in which case multiple imputation will not have led to bias in the results^69^. When looking at the data, a combination of the two reasons seems plausible. Furthermore, there is less missing data in the UKZN dataset but the UKZN questionnaire is smaller than the UoA one. Social disability bias might play a role since there is missingness in ‘difficult’ questions. Additionally, there are inconsistencies in the literature regarding the optimal cut-off score to use for the AUDIT-C. We have used a cut-off point of ≥ 5 to determine hazardous drinking in male students and a score of ≥ 4 for female students. These cut-off points were based on a previous study which stated that among a tertiary student population, in which heavy alcohol use is normative behaviour, less strict cut-off points for the AUDIT-C should be used to detect hazardous drinking behaviour^35^. But in other studies, the cut-off points are ≥ 3 for women and ≥ 4 for men^36^, are usually used.

A strength of the current study is that despite the retrospective use of the dataset, the sample size was large enough to detect significant values. All participants that were already recruited, were included in the analyses to increase the power further. Moreover, previous studies set in South Africa suggest that due to both the high prevalence of HIV and crime and the fact that students often have to overcome poor academic preparation before starting their university studies implies that the social, health and academic consequences of harmful drinking might be worse for students in a developing country such as South Africa than they would be in a developed country like Belgium^67^. This contradicts the findings from this study and further research is therefore needed to assess factors related to hazardous drinking in developing countries.

## Conclusion

Results from this study indicate that alcohol consumption is higher among Belgian students from the UoA compared to South African from the UKZN. Not only, do UoA students consume alcohol more often their risk for hazardous drinking is also higher compared to UKZN students. Multivariate logistic regression was used to assess factors relating to hazardous drinking for male- and female students of both universities separately. Risk factors differed between the genders, with risk factors identified for males being; living independently, being part of a youth movement or a board of a student club, drinking beer or hard liquors and smoking tobacco. While for females, an increase in age, sports club membership, drinking wine or hard liquor, smoking tobacco or cannabis and used illegal drugs were risk factors for hazardous drinking. These findings suggest that factors for hazardous drinking diverge between the genders. More research is needed to understand why determinants of hazardous drinking differ between male- and female students. But primarily research assessing differences in hazardous drinking between male and female students from developed and developing countries is needed to gain further insight in not only difference between gender but also in the cultural and ethnic factors behind them.
